# Herpes zoster vaccination uptake in rheumatic diseases: persistent implementation gap despite improved uptake

**DOI:** 10.1093/rap/rkag090

**Published:** 2026-07-17

**Authors:** Marco Krasselt

**Affiliations:** Rheumatology, Medical Clinic III: Endocrinology, Nephrology and Rheumatology, Department of Internal Medicine, Neurology and Dermatology, University of Leipzig Medical Centre, Leipzig, Germany

Key message

- Only one in three patients with inflammatory rheumatic diseases is vaccinated against herpes zoster (33.5%).

Dear Editor, Patients with inflammatory rheumatic diseases (IRDs) face a substantially elevated risk of herpes zoster (HZ) and its complications, driven by underlying immune dysregulation and immunosuppressive therapy [1, 2]. International guidelines, including the 2022 ACR Guideline for Vaccinations in Patients with IRDs [3] and the 2019 EULAR recommendations (with ongoing updates) [4], strongly advocate recombinant zoster vaccine (RZV) use in this population. Earlier German real-world data nevertheless documented persistently low HZ vaccination rates (13.1% in 2023) [5]. US claims data indicate that shortly after RZV licensure only 14.8% of commercially insured and 43.2% of Medicare beneficiaries age ≥50 years with immune-mediated inflammatory diseases had received at least one RZV dose in 2018–2019 [6]. Recent Israeli nationwide data showed even lower coverage (3.6%) [7]. Therefore the aim of this study was to obtain current HZ vaccination rates in a German outpatient clinic.

HZ vaccination status was evaluated from December 2024 to May 2025 in consecutive adult IRD patients scheduled for routine consultation. Group differences in HZ vaccination status were analysed using chi-squared or Fisher’s exact tests, as appropriate. Independent associations were examined with multivariable logistic regression. A two-sided *P*-value <0.05 was considered statistically significant.

HZ vaccination was defined as documentation of at least one dose of RZV. Prior HZ episodes were assessed by patient-reported history and verified from the medical records when available.

All procedures performed in this survey were in accordance with the ethical standards of the institutional and/or national research committee and with the 1964 Helsinki Declaration and its later amendments or comparable ethical standards. The local ethics committee of the medical faculty of Leipzig University approved the design of the study (061-25-ek). Informed consent was obtained from all individual participants included in the study.

The sample included 272 IRD patients [median age 61.5 years (range 18–90), 63% female; 45% RA, 29% SpA/PsA, 14% CTDs including SLE and 6% vasculitis]. The rest of the patient characteristics are presented in Supplementary Table S1. Overall HZ vaccination coverage reached 33.5% (91/272), with 89% of vaccinated patients having completed the full two-dose RZV schedule. A total of 35% (95/272) of the cohort reported at least one prior HZ episode. Paradoxically, vaccination uptake was numerically lower in patients who had already experienced HZ (28.4% *vs* 36.2%; *P* = 0.226), although the difference did not reach statistical significance. There was no difference in vaccination regarding the underlying IRD.

In multivariable logistic regression {adjusted for age, diagnosis, medication [conventional synthetic DMARDs, biologic DMARDs, immunosuppressives, Janus kinase (JAK) inhibitors, prednisone] and co-vaccination status against influenza and pneumococcus}, the following independent associations emerged (Table 1): age ≥60 years [adjusted odds ratio (aOR)] 5.18 (95% CI 3.01, 8.92), *P* < 0.001], JAK inhibitor use [aOR 5.17 (95% CI 1.24, 21.55), *P* = 0.024], pneumococcal vaccination [aOR 6.31 (95% CI 3.03, 13.15), *P* < 0.001) and influenza vaccination [aOR 2.20 (95% CI 1.03, 4.68), *P* = 0.041). For the detailed results of the univariate analysis see Supplementary Table S1.

**Table 1:** Multivariable logistic regression analysis for HZ vaccination uptake.

Variable	aOR (95% CI)	*P*-value
Age ≥60 years	5.18 (3.01, 8.92)	<0.001
JAK inhibitor use	5.17 (1.24, 21.55)	0.024
Pneumococcal vaccination	6.31 (3.03, 13.15)	<0.001
Influenza vaccination	2.20 (1.03, 4.68)	0.041
Diagnosis: SpA/PsA (*vs* RA)	0.89 (0.48, 1.65)	0.712
Diagnosis: SLE/CTD (*vs* RA)	1.12 (0.59, 2.13)	0.721
Diagnosis: vasculitis/other (*vs* RA)	0.76 (0.31, 1.85)	0.544
csDMARD use	1.08 (0.62, 1.88)	0.782
bDMARD use	0.94 (0.51, 1.73)	0.845
Rituximab use	0.67 (0.22, 2.01)	0.475
Prednisone use	1.35 (0.78, 2.34)	0.284
Other immunosuppressives	1.21 (0.67, 2.19)	0.529

bDMARD: biologic DMARD; csDMARD: conventional synthetic DMARD.

Values are shown for every covariate included in the model.

HZ vaccination coverage in the analysed cohort (33.5%) was higher than in previous German real-world cohorts [5] and a recent international study [7]. Age-stratified rates (14.4% in patients <60 years of age and 48.1% in patients ≥60 years of age) were comparable to those observed in US claims data shortly after RZV licensure (14.8% in commercially insured patients age 50–64 years and 43.2% in Medicare beneficiaries age ≥65 years [6]. Interestingly, patients with SLE (who carry one of the highest risks of HZ among patients with IRD) did not show higher vaccination rates than the overall cohort. In Germany, the Standing Committee on Vaccination (STIKO) indication was expanded in November 2025 from the previous age threshold of 50 years to immunocompromised adults ≥18 years, with routine reimbursement following in February 2026 [8]. The absence of an official recommendation and reimbursement for patients <60 years of age throughout the entire data collection period is therefore likely to have contributed substantially to the low vaccination uptake observed in the younger cohort.

HZ vaccination uptake remains substantially low despite strong recommendations by current ACR and EULAR guidelines for this high-risk population [3, 4]. A substantial implementation gap persists, especially in younger patients and, paradoxically, among those with a history of HZ.

Strong clustering with pneumococcal and influenza vaccination points to a general ‘vaccination-mindedness’ or better integrated care pathways rather than disease- or therapy-specific barriers. The independent positive association with JAK inhibitor use likely reflects heightened physician awareness in this very high-risk group, consistent with recent data on RZV efficacy and safety under immunosuppression. These findings align with the good immunogenicity and safety of RZV even under various immunosuppressive regimens in IRDs [2]. This single-centre, cross-sectional study is subject to potential selection bias and recall bias regarding prior HZ episodes. The multivariable analyses are exploratory given the moderate sample size, thus confirmation in larger multicentre cohorts is needed.

Taken together, these results underline the urgent need for systematic strategies: routine vaccination status assessment at every rheumatology visit, electronic reminders, co-administration with seasonal influenza/pneumococcal vaccines and targeted education for both patients and physicians. Without such measures, preventable HZ cases and complications will continue to burden this vulnerable population.

## Supplementary Material

rkag090_Supplementary_Data

## Data Availability

The data underlying this article will be shared upon reasonable request to the corresponding author.
