# Supplementary material for: Herpes zoster vaccination uptake in rheumatic diseases: persistent implementation gap despite improved uptake
Source: Rheumatol Adv Pract. 2026 Jul 17;10(3):rkag090. doi: 10.1093/rap/rkag090 (PMC13441164; doi:10.1093/rap/rkag090)
Supplement: Supplementary file 1 [file rkag090_supplementary_data.docx]

**Supplementary Table S1. Univariate HZ vaccination coverage**

**stratified by key determinants.**

| **Characteristic** | **n** | **HZ vaccinated n (%)** | **p-value** |
| --- | --- | --- | --- |
| **Overall** | 272 | 91 (33.5) | – |
| **Age ≥60 years** | 154 | 74 (48.1) | <0.001 |
| **Pneumococcal vaccination** | 143 | 77 (53.8) | <0.001 |
| **Influenza vaccination** | 178 | 77 (43.3) | <0.001 |
| **Prior HZ episode** | 95 | 27 (28.4) | 0.226 |
| **JAK inhibitor use** | 11 | 6 (54.5) | 0.189 |

HZ = herpes zoster; JAK = Janus kinase; p-values refer to the comparison

of the respective category versus the complementary group (e.g., age <60

vs. ≥60 years; prior HZ yes vs. no).
